# Immunotherapy and fatigue: what we know and what we don’t know

**DOI:** 10.18632/oncotarget.27946

**Published:** 2021-04-13

**Authors:** Muhammad Azeem Khan, Vaia Florou, Umang Swami

**Keywords:** fatigue, PD-1, CTLA-4, testosterone, immunotherapy-related fatigue

***News on:** Testosterone deficiency in men receiving immunotherapy for malignant melanoma by Peters et al. Oncotarget. 2021; 12:199–208. https://doi.org/10.18632/oncotarget.27876. [PubMed]*

Cancer cells can hijack the immune checkpoint signaling pathway to evade cytotoxic T-cell mediated cell death. The introduction of immune checkpoint inhibitors (ICI), which disrupt these immune evasion mechanisms, has improved the prognosis of patients with numerous advanced malignancies [1]. Despite their benefits, ICIs are associated with toxicities that arise in part from enhanced immune activity [1]. Immune-related adverse events usually occur within the first few weeks to months of the start of treatment but are known to occur any time, even after treatment discontinuation [2]. One of the most common adverse effects of ICI is fatigue. In clinical studies, the incidence of fatigue was 16–37% with single anti-PD1 agents and 12–24% with anti-PD-L1 agents. The combination of anti-PD-1 and anti-PD-L1 agents with conventional or targeted anti-cancer therapies was found to result in an even higher incidence of fatigue, ranging from 21 to 71% [3]. In a phase III trial of ipilimumab, an anti-cytotoxic T-lymphocyte-associated protein 4 (CTLA-4) agent, the incidence of fatigue was 40% [4]. For most patients, symptoms of fatigue were mild and not associated with other constitutional symptoms. No direct association between the dose of ICI and fatigue has been found, and currently, the exact mechanism of treatment-related fatigue is unknown [4]. However, in some patients, fatigue may be the manifestation of other known adverse effects of immune checkpoint blockade such as cardiac, pulmonary, or endocrine toxicity.

In a recent single-institution retrospective study Peters et al. [5], investigated men with malignant melanoma who received immunotherapy and had low testosterone levels. From the overall cohort of 143 male patients with melanoma, the authors identified 49 patients who were treated with immunotherapy and at least twice underwent testosterone evaluations. Of these 43 patients reported fatigue, and 34 had low testosterone (defined as two lab values with testosterone < 300 ng/dl). Only three patients received testosterone replacement therapy and they did have an improvement in fatigue. However, they were also treated with steroids and thyroid replacement therapy. Though any direct inference or association cannot be deduced from these results, this study highlights the fact that there exists a wide variability among treating physicians with regards to when and what work-up is ordered for fatigue, how the results are interpreted, and what management options are employed. Within the same institution, there may exist a heterogeneity in practice patterns with regards to evaluation and treatment of fatigue for patients on immunotherapy. These findings emphasize the need for implementation and rigorous adherence of standardized, evidence-based guidelines for the management of fatigue due to immunotherapy in patients to improve their overall quality of life.

Fatigue is not only the most common and disruptive effect of ICI but is also one of the hardest to treat. Apart from being caused by immune checkpoint inhibitors, fatigue can result from concomitant systemic therapies such as cytotoxic chemotherapy, targeted agents, and radiation. Fatigue can also be an effect of comorbidities or cancer itself. The precise biological mechanism by which ICI and cancer cause fatigue is unclear and is an area of active investigation. Anemia, cytokine dysregulation, disruption of the hypothalamic-pituitary-adrenal axis, neurotransmitter disruption, and changes in adenosine triphosphate and muscle metabolism have been implicated in studies [6]. Interestingly, certain circulating inflammatory cytokines, have been shown to mediate a sense of tiredness and fatigue by modulating the signaling processes in the central nervous system and may be responsible for the underlying mechanism for immunotherapy-related fatigue. Cancer itself can produce such inflammatory cytokines or they can be produced because of the body’s response to cancer or treatment. For example, radiation and chemotherapy lead to tissue damage, which then produces the same inflammatory cytokines causing fatigue and exhaustion [7].

The National Comprehensive Cancer Network (NCCN) has published guidance for the management of immunotherapy-related toxicities which includes recommendations for the evaluation of immunotherapy-related fatigue [8]. The NCCN panel recommends obtaining a history and physical with a full set of vital signs and conducting a medication review. The recommended laboratory workup consists of complete blood count, complete metabolic profile, as well as laboratory screening for endocrine dysfunction with a thyroid-stimulating hormone, free thyroxine level, and morning cortisol, ACTH (if cortisol level is subnormal), and testosterone in males. If grade 3 or 4 fatigue (not relieved by rest, resulting in limited self-care) is present, the NCCN panel recommends holding or discontinuing immunotherapy if a reversible cause of fatigue cannot be found.

Data from prospective research on fatigue in patients receiving immunotherapy are lacking. NCT03525873 is an ongoing, randomized phase III trial comparing the stimulant methylphenidate plus physical activity to placebo plus physical activity in reducing cancer-related fatigue in patients with metastatic cancer receiving anti-PD-1 agents. Gaining an understanding of the specific mechanisms that cause immunotherapy-related fatigue is vital to develop strategies to mitigate it. Possible ways to gain such insights would involve analyzing stored serum or plasma samples collected from large registration trials for changes in cytokines and correlation with patient-reported fatigue. Recently gut microbiome has been associated with fatigue [9] and it will be interesting to explore its role in ICI-related fatigue. There is also a need to investigate various approaches to help mitigate the burden of fatigue including cytokine antagonists, other pharmacotherapies such as methylphenidate, and interventions such as cognitive-behavioral therapies, mind-body therapies, and supervised exercise programs [6, 7]. Any success in alleviating ICI-related fatigue will have a tremendous impact on the quality of life of countless patients with cancer.
